# The first step in creating national Chronic Kidney Disease (CKD) guidelines – a questionnaire

**DOI:** 10.11613/BM.2019.030301

**Published:** 2019-08-05

**Authors:** Vanja Radišić Biljak, Anders Grubb, Isabel Cachapuz Guerra, Etienne Cavalier, Stella Raymondo, Rosa Sierra-Amor, Michele Mussap, Shanthi Naidu Kamathan, Dogan Yucel, Pradip Datta, Takashi Wada, Flavio F. Alcantara

**Affiliations:** 1 Department of Medical Laboratory Diagnostics, University Hospital „Sveti Duh“, Zagreb, Croatia; 2 Department of Clinical Chemistry, Skåne University Hospital, Lund, Sweden; 3 Department of Clinical Pathology, Pedro Hispano Hospital – Matosinhos Local Health Unit, Portugal; 4 CHU de Liège, University of Liège, Domaine du Sart-Tilman, Liège, Belgium; 5 Committee of Standardization and Quality Control, Faculty of Chemistry, University of Uruguay, Montevideo, Uruguay; 6 Laboratorio LAQUIMS, S.C., Veracruz, Ver., México; 7 Laboratory Medicine, Department of Surgery, School of Medicine, University of Cagliari, Cagliari, Italy; 8 Department of Laboratory Medicine, Care Hospital, Banjara Hills, Hyderabad, India; 9 Department of Medical Biochemistry, Ankara Training nand Research Hospital, University of Health Sciences, Ankara, Turkey; 10 Siemens Healthineers, Newark, Delaware, USA; 11 Department of Nephrology and Laboratory Medicine, Kanazawa University, Japan; 12 Central Laboratory Division Department of Clinical Pathology, Clinical Hospital University of Sao Paulo – Medical School, Sao Paulo, Brazil; 13 Instituto de Analises Clínicas de Santos (IACS), Santos, Brazil

**Keywords:** chronic kidney disease (CKD), International Federation of Clinical Chemistry and Laboratory Medicine (IFCC), guidelines, action plan, questionnaire

Chronic kidney disease (CKD), defined as decreased kidney function shown by glomerular filtration rate (GFR) of less than 60 mL/min/1.73m^2^, or markers of kidney damage, or both, of at least 3 months duration, regardles of underlying cause, is increasingly recognised as a global public health problem (*1*, *2*). In 2016 the International Society of Nephrology identified key activities for the next 5-10 years to create an action plan for determining and monitoring the prevalence of chronic kidney disease (*2*, *3*). Some of the proposed ten themes include strenghtening CKD surveillance and establishing better diagnostic methods in CKD where our role as clinical chemists is clearly emphasized.

Identification of CKD relies primarily on serum creatinine measurements and equations to estimate GFR and measurement of creatinine and albuminuria are of utmost importance for diagnosis and classification of CKD. According to the 2017 action plan, the first step in improving CKD monitoring activities includes strenghtening CKD surveillance, partly by achieveing a uniform measurement of CKD markers and ensuring that wherever serum creatinine is measured, estimated GFR is reported. Additionally, there should be increasing efforts to use albuminuria and eGFR in combination, whenever serum creatinine is measured. At the same time, healthcare providers and clinical chemists should work towards global implementation of the the diagnosis and staging of CKD on the basis of measuring or estimating glomerular filtration rate and measuring albuminuria (*2*).

One of the possible solutions to achieveing this pre-set goals is to continue to develop, update, and improve clinical practice guidelines (CPGs) pertinent to CKD on a global scale (*2*). It is evident that CPGs relating to laboratory diagnostic testing are increasingly produced with the aim of standardizing practice and improving patient care based on the best available evidence (*4*). Perhaps the initial step to create a guideline is to explore the current status of CKD testing in a national enviroment, which has been carried out successfully in some European countries (*5*, *6*).

In order to actively pursue the solutions to the described action plan, we as the International Federation of Clinical Chemistry and Laboratory Medicine (IFCC) Committee on Kidney Disease – C-KD (previously Committee on Chronic Kidney Disease – C-CKD) decided to provide assistance where required for member organizations and others in planning and implementing CKD testing policies and guidelines, which is in accordance with our Committee objectives (*7*). Therefore, to help the National Societies across the Globe with the assessment process of exploring the current status of CKD testing, we have prepared a universal questionnaire that can be used as a starting point in the process (Table 1). The questionnaire is at the most part constructed from the already successfully performed Croatian and Portuguese surveys (*8*, *9*). The Croatian survey results set the background for the creation of the first national Croatian CKD recommendations (*8*, *10*).

**Table 1:** A proposed IFCC C-KD universal questionaire

**Questions**	**Answers**
**Serum creatinine**	
1. Does your laboratory measure serum creatinine?	yes no
2. Please specify your method for measuring serum creatinine (more than one may be selected)	uncompensated Jaffe compensated Jaffe, IDMS calibrated enzymatic method aligned with IDMS method whole blood creatinine dry chemistry other (please specify)
3. Specify your reagent manufacturer(s)	
4. Specify your type of instrument(s)	
5. In which units the serum creatinine results are reported?	mg/dL µmol/L both
6. Specify reference interval for adults used for serum creatinine in your laboratory	
**eGFR_creatinine_**	
7. Do you automatically report the results of estimated Glomerular Filtration Rate (eGFR_creatinine_) along with the serum creatinine concentration?	yes, automatically with every result of serum creatinine yes, whenever ordered by the physician no
8. Do you consider reporting the eGFR_creatinine_?	yes no
9. Which equation do you use for estimating GFR for routine reporting (more than one may be selected)	MDRD-4 equation for non-standardized creatinine MDRD-4 equation for standardized creatinine MDRD-6 CKD-EPI equation Cockroft-Gault other (please specify) I don’t know
10. At what value do you point to an eGFR_creatinine_ value higher than (>)?	we report all values for eGFR_creatinine_ as the exact number we report values above 60 mL/min/1.73m^2^ as > 60 mL/min/1.73m^2^ we report values above 90 mL/min/1.73m^2^ as > 90 mL/min/1.73m^2^ other (please specify)
11. How do you report the results concerning the parameter „race“?	two results, „Black“ and „White“ race evaluated during the appointment patient is asked to report its race „Race“ parameter is not included other (please specify)
12. In the eGFR_creatinine_ result, there are included observations and possible variations of this value with (multiple answers possible):	nutritional status pregnancy admitted patients we don’t include observations about possible variations of eGFR other(s) (please specify)
13. Special situations: in-patients	we report eGFR_creatinine_ for in-patients we don’t report eGFR_creatinine_ for in-patients
14. Which equations/formulas do you use to estimate the GFR in children?	Schwartz equation (original) Schwartz equation (modified) other (please specify) we don’t calculate eGFR_creatinine_ for children
**Serum (plasma) cystatin C**	
15. Does your laboratory measure serum cystatin C?	yes no
16. Please specify your method for measuring serum cystatin C (more than one may be selected)	immunoturbidimetry immunonephelometry other (please specify)
17. Specify your method(s) traceability	
18. Specify your reagent manufacturer(s)	
19. Specify your type of instrument (s)	
20. In which units the serum cystatin C results are reported?	mg/L other (please specify)
21. Specify reference interval for adults used for serum cystatin C in your laboratory	
**eGFR_cystatin C_**	
22. Do you automatically report the results of estimated Glomerular Filtration Rate based upon cystatin C (eGFR_cystatin C_) along with the serum cystatin C concentration?	yes, automatically with every result of serum cystatin C yes, whenever ordered by the physician no
23. Do you consider reporting the eGFR_cystatin C_?	yes no we already report it
24. Which eGFR_cystatin C_-equation for adults do you use for routine reporting (more than one may be selected)	CAPA CKD-EPI_cystatin C_ other (please specify)
25. At what value do you point to an eGFR_cystatin C_ value higher than (>)?	we report all values for eGFR_cystatin C_ as the exact number we report values above 60 mL/min/1.73m^2^ as > 60 mL/min/1.73m^2^ we report values above 90 mL/min/1.73m^2^ as > 90 mL/min/1.73m^2^ other (please specify)
26. Which eGFR_cystatin C_-equation do you use to estimate the GFR in children?	CAPA CKD-EPI_cystatin C_ other (please specify) we don’t estimate GFR for children
**Urine protein and albumin**	
27. Do you perform the following tests?	urine protein urine albumin both none of those
28. Which is recommended sample for measuring urine protein?	24 hour urine sample random urine sample first morning urine sample not specified other (please specify)
29. Please specify your method for measuring urine protein	nephelometry turbidimetry colorimetry dry chemistry other (please specify)
30. Specify your method(s) traceability	
31. Specify your reagent manufacturer(s)	
32. Specify your type of instrument(s)	
33. How do you report urine protein results (more than one may be selected)?	mg/mmol creatinine mg/g creatinine g/24h g/L
34. Please specify your reference interval (cut-off value) for urine protein	
35. Which is recommended sample for measuring urine albumin?	24 hour urine sample random urine sample first morning urine sample not specified other (please specify)
36. Please specify your method for measuring urine albumin	nephelometry turbidimetry colorimetry dry chemistry other (please specify)
37. Specify your method(s) traceability	
38. Specify your reagent manufacturer(s)	
39. Specify your type of instrument(s)	
40. How do you report urine albumin results (more than one may be selected)?	mg/mmol creatinine mg/g creatinine μg/min mg/24h μg/mL mg/L
41. Please specify your reference interval (cut-off value) for urine albumin	
IDMS - isotope dilution mass spectrometry. MDRD - modification of diet in renal disease. CKD-EPI - chronic kidney disease epidemiology collaboration. CAPA - Caucasian, Asian, pediatric and adult.	

To facilitate the usage of the questionnaire, we have translated it to some of the most prevalent languages (Tables 2-9). It is important to emphasize that the questionnaire should be used in accordance with the current situation in particular countries. In other words, we encourage the future users to make as many amendments as necessary, either shortening the proposed list of questions or adding some questions that are not in the questionnaire, but that are considered useful.

**Table 2:** A proposed IFCC C-KD universal questionaire – German translation

**Fragen**	**Antworten**
**Kreatinin**	
1. Misst ihr Labor Kreatinin?	Ja Nein
2. Bitte geben sie die Messmethode für die Kreatininbestimmung an (Mehrfachantworten möglich)	Unkompensierte Jaffe Methode Kompensierte Jaffe Methode, IDMS kallibriert Enzymatische Methode abgestimmt auf die IDMS Methode Vollblutmethode Trockenchemisch Andere Methode (Bitte angeben) Weiß nicht
3. Bitte geben sie den Reagenzhersteller des Kreatinin-Assays an	
4. Bitte geben sie das Gerät an auf welchem sie Kreatinin messen	
5. Mit welchen Einheiten geben sie Kreatininwerte auf ihren Befunden an?	mg/dL µmol/L beides
6. Bitte geben sie das Referenzintervall/Normalwert an, welches für den Kreatininwert bei Erwachsenen verwenden	
**eGFR_creatinine_**	
7. Geben sie automatisch den entsprechenden Wert der errechneten estimated Glomerular Filtration Rate (eGFR_creatinine_) am Befund mit an?	Ja, automatische Berechnung bei jeder Kreatinin-Bestimmung Ja, wenn angefordert Nein
8. Erwägen sie die eGFR_creatinine_ am Befund mit anzugeben?	Ja Nein
9. Welche Formel verwenden sie für die Berechnung der eGFR_creatinine_ bei Erwachsenen auf Routinebefunden? (Mehrfachantworten möglich)	MDRD-4 Formel für nicht-standardisiertes Kreatinin MDRD-4 Formel für standardisiertes Kreatinin MDRD-6 CKD-EPI Cockroft-Gault Andere Formel (Bitte angeben)
10. Ab welchem Messwert der eGFR_creatinine_ geben Sie Werte mit „größer als“ (>) an?	Wir geben alle Werte exakt so an wie sie berechnet wurden. Wir geben Werte über 60 mL/min/1.73m^2^ als > 60 mL/min/1.73m^2^ an Wir geben Werte über 90 mL/min/1.73m^2^ als > 90 mL/min/1.73m^2^ an Anderes (bitte angeben)
11. Wie geben sie Kreatinin-Werte auf dem Befund an in Bezug auf den Parameter „ethnische Herkunft“	Es gibt zwei Ergebnisse, „Schwarz“ und „Weiss“ Die ethnische Herkunft wird während des Blutabnahmetermins bestimmt Der Patient muss seine ethnische Herkunft angeben Die ethnische Herkunft wird nicht berücksichtigt Anderes (bitte angeben)
12. In der Beurteilung des eGFR_creatinine_ Wertes sind folgende Einflußfaktoren berücksichtigt (Mehrfachantworten möglich):	Ernährungsstatus Schwangerschaft Staionäre Patienten Es werden (können) keine Einflußfaktoren berücksichtigt (werden) Anderes (bitte angeben)
13. Besondere Situationen: Stationäre Patienten	Wir geben die eGFR_creatinine_ auch für stationäre Patienten an Wir geben die eGFR_creatinine_ für stationäre Patienten nicht an
14. Welche Formel verwenden sie für die Berechnung der eGFR_creatinine_ bei Kindern auf Routinebefunden?	Schwartz Formel (original) Schwartz Formel (modifiziert) Anderes (bitte angeben) Wir berechnen die eGFR_creatinine_ nicht bei Kindern
**Cystatin C**	
15. Wird in ihrem Labor Cystatin C bestimmt?	Ja Nein
16. Bitte geben sie die Messmethode für die Cystatin C-Bestimmung an (Mehrfachantworten möglich)	Immunturbidimetrie Immunnephelometrie Andere Methode (bitte angeben)
17. Bitte geben sie die Rückverfolgbarkeit („traceability“) ihrer Methode und deren Kalibration an	
18. Bitte geben sie den Reagenzhersteller des Cystatin C-Assays an	
19. Bitte geben sie das Gerät an auf welchem sie Cystatin C messen	
20. Mit welchen Einheiten geben sie Cystatin C-Werte auf ihren Befunden an?	mg/L Anderes (bitte angeben)
21. Bitte geben sie das Referenzintervall/Normalwert an, welches für den Cystatin C-Wert bei Erwachsenen verwenden	
eGFR_cystatin C_	
22. Geben Sie automatisch mit dem Cystatin C Ergebnis die Cysatatin C basierte glomeruläre Filtrationsrate (eGFR_cystatin C_) mit an?	Ja, immer mit jedem Cystatin C Ergebnis Ja, wenn vom Arzt angefordert nein
23. Planen Sie die eGFR_cystatin C_ am Befund anzugeben?	Ja nein wir geben sie bereits an
24. Welche eGFR_cystatin C_ Gleichung für Erwachsene verwenden Sie für Routine-Befunde (Mehrfachnennung möglich)	CAPA CKD-EPI_cystatin C_ andere (bitte angeben)
25. Ab welchem Messwert der eGFR_cystatin C_ geben Sie Werte mit größer als (>) an?	Wir geben alle Messwerte der eGFR_cystatin C_ als exakten Zahlen wert an Wir geben Messwerte über 60 mL/min/1.73m^2^ an als > 60 mL/min/1.73m^2^ Wir geben Messwerte über 90 mL/min/1.73m^2^ an als > 90 mL/min/1.73m^2^ andere (bitte angeben)
26. Welche eGFR_cystatin C_-Gleichung verwenden Sie für Kinder zur Abschätzung der GFR?	CAPA CKD-EPI_cystatin C_ andere (bitte angeben) Wir berechnen keine GFR für Kinder
**Gesamteiweiß und Albumin im Urin**	
27. Führen Sie die folgenden Untersuchungen durch?	Gesamteiweiß im Urin Albumin im Urin beide Keine von beiden
28. Was ist das empfohlene Probenmaterial für die Gesamteiweißbestimmung im Urin?	24 Stunden Sammelurin Spontanurin Erster Morgenurin Nicht spezifiziert andere (bitte angeben)
29. Bitte geben Sie Ihre Methode für die Gesamteiweißbestimmung im Urin an	Nephelometrie Turbidimetrie Colorimetrie Trockenchemie andere (bitte angeben)
30. Bitte geben sie die Rückverfolgbarkeit („traceability“) ihrer Methode und deren Kalibration an	
31. Bitte geben sie den Reagenzhersteller des Gesamteiweiß im Urin-Assays an	
32. Bitte geben sie das Gerät an auf welchem sie Gesamteiweiß im Urin messen	
33. Wie geben Sie Gesamtweiß im Urin – Ergebnisse an? (Mehrfachnennung möglich)	mg/mmol Kreatinin mg/g Kreatinin g/24h g/L
34. Bitte geben Sie Ihren Referenzbereich für das Gesamteiweiß im Urin an	
35. Was ist das empfohlenen Probenmaterial für die Albuminbestimmung im Urin?	24 Stunden Sammelurin Spontanurin Erster Morgenurin Nicht spezifiziert andere (bitte angeben)
36. Bitte geben Sie Ihre Methode für die Albuminbestimmung im Urin an	Nephelometrie Turbidimetrie Colorimetrie Trockenchemie andere (bitte angeben)
37. Bitte geben sie die Rückverfolgbarkeit („traceability“) ihrer Methode und deren Kalibration an	
38. Bitte geben sie den Reagenzhersteller des Gesamteiweiß im Urin-Assays an	
39. Bitte geben sie das Gerät an auf welchem sie Gesamteiweiß im Urin messen	
40. Wie geben Sie Albumin im Urin – Ergebnisse an? (Mehrfachnennung möglich)	mg/mmol Kreatinin mg/g Kreatinin μg/min mg/24h μg/mL mg/L
41. Bitte geben Sie Ihren Referenzbereich für Albumin im Urin an	
IDMS - isotope dilution mass spectrometry. MDRD - modification of diet in renal disease. CKD-EPI - chronic kidney disease epidemiology collaboration. CAPA - Caucasian, Asian, pediatric and adult.	

**Table 3:** A proposed IFCC C-KD universal questionaire – French translation

**Questions**	**Réponses**
**Créatinine sérique ou plasmatique**	
1. Votre laboratoire dose-t-il la créatinine sérique?	Oui Non
2. Quelle méthode utilisez-vous (plusieiurs réponses possibles)?	Jaffe Non compensé Jaffe compensé, standardisé IDMS Méthode enzymatique, standardisée IDMS Créatinine sur sang complet Chimie sèche Autre (sépcifier)
3. De quelle(s) compagnie(s) proviennent les réactifs?	
4. Quel automate utilisez-vous?	
5. Quelles sont vos unités pour la créatinine sérique?	mg/dL µmol/L les deux
6. Quelles sont vos valeurs de référence chez l’adulte?	
**Estimation du Débit de filtration glomérulaire (eDFG créatinine)**	
7. Fournissez-vous automatiquement le eDFG avec le dosage de la créatinine?	Oui, systématiquement avec le dosage de la créatinine Oui, lorsque c’est demandé par le médecin prescripteur Non
8. Si vous avez répondu non à la question précédente, envisagez-vous de fournir le eDFG?	Oui Non
9. Quelle(s) équation(s) utilisez-vous en routine pour le eDFG? (plusieurs réponses possibles)?	MDRD-4 paramètres pour créatinine Non-standardisée IDMS MDRD-4 paramètres pour créatinine standardisée IDMS MDRD-6 paramètres CKD-EPI Cockcroft-Gault Autre (précisez) Je ne sais pas
10. A partir de quelle valeur tronquez-vous le résultat de l’eDFG (> à)?	Nous ne tronquons pas les résultats Nous tronquons au-delà de 60 mL/min/1.73m^2^ Nous tronquons au-delà de 90 mL/min/1.73m^2^ Autre (spécifier)
11.Comment gérez-vous le paramètre „race“?	2 résultats, „Noir“ et „Blanc“ Donnée obtenue lors de la prise de sang On demande au patient de préciser sa race Nous n’incluons pas le paramètre „race“ Autre (spécifiez)
12. Lorsque que vous fournissez le eDFG, vous donnez des comentaires sur des variations possibles liées à:	Statut nutritionnel Grossesse Patient hospitalisé Nous ne fournissons pas de commentaires sur des variations possbles du DFG Autre(s) (spécifiez)
13. Siutuation particulière des patients hospitalisés	Nous fournissons le eDFG chez les patients hospitalisés Nous ne fournissons pas le eDFG chez les patients hospitalisés
14. Avec quelle(s) équation(s) estimez-vous le DFG chez les enfants?	Schwartz (originale) Schwartz (modifiée) Autre (spécifiez) Nous ne calculons pas le DFG chez les enfants
**Cystatin C sérique (plasmatique)**	
15. Votre laboratoire mesure-t-il la CysC?	Oui Non
16. Quelle méthode utilisez-vous? (plusieurs réponses possible)	Immunoturbidimétrie Immunonéphélométrie Autre (spécifiez)
17. Spécifiez la traçabilité de votre méthode	
18. Quel est le fabricant du réactif?	
19. Quel est le fabricant de l’instrument (ou des instruments) que vous utilisez pour doser la CysC?	
20. En quelle unité rapportez-vous la CysC sérique?	mg/L Autre (spécifiez)
21. Quelles sont les valeurs de références pour adultes que vous utilisez ?	
**eDFG_cystatin_**_C_	
22. Fournissez-vous automatiquement une estimation du DFG basée sur la CysC lorsqu’un dosage de CysC est demandé?	Oui, automatiquement Oui, si demandé par le médecin-prescripteur Non
23. Avez-vous l’intention de fournir l’estimation du DFG basée sur la CysC?	Oui Non Nous la fournissons déjà
24. Quelle équation utilisez-vous pour reporter le DFG basé sur la CysC? (plusieurs réponses possibles)	CAPA CKD-EPI_cystatin C_ Autre (spécifiez)
25. A partir de quelle valeur tronquez-vous le résultat de l’eDFG basé sur la CysC (> à)?	Nous ne tronquons pas les résultats Nous tronquons au-delà de 60 mL/min/1.73m^2^ Nous tronquons au-delà de 90 mL/min/1.73m^2^ Autre (spécifier)
26. Avec quelle(s) équation(s) estimez-vous le DFG chez les enfants?	CAPA CKD-EPI_cystatin C_ Autre (spécifiez) Nous ne calculons pas le DFG chez les enfants
**Protéinurie et albuminurie**	
27. Réalisez-vous ces dosages en routine?	Protéines urinaires Albumine urinaire Tous les deux Aucun des deux
28. Quel échantillon recommandez-vous pour le dosage des protéines urinaires?	Urines de 24 heures Echantillon urinaire aléatoire Premières urines du matin à jeun Aucune recommandation Autres (spécifiez)
29.Quelle méthode utilisez-vous pour doser les protéines urinaires?	Néphélométrie Turbidimétrie Colorimétrie Chimie sèche Autre (spécifiez)
30. Quelles est la traçabilité de vos méthodes?	
31. De quelle compagnie proviennent les réactifs?	
32. Quel instrument utilisez-vous?	
33. Quelles sont vos unités pour le dosage des protéines urinaires (plusieurs réponses possibles)?	mg/mmol créatinine mg/g créatinine g/24h g/L
34. Quels sont vos valeurs de référence (cut-off) pour la protéinurie?	
35. Quel échantillon recommandez-vous pour le dosage de l’albumine urinaire?	Urines de 24 heures Echantillon urinaire aléatoire Premières urines du matin à jeun Aucune recommandation Autres (spécifiez)
36. Quelle méthode utilisez-vous pour le dosage de l’albumine urinaire?	Néphélométrie Turbidimétrie Colorimétrie Chimie sèche Autre (spécifiez)
37. Quelle est la traçabilité de votre méthode?	
38. De quelle compagnie proviennent les réactifs?	
39. Quel instrument utilisez-vous?	
40. Quelles sont vos unités pour le dosage de l’albumine urinaire (plusieurs réponses possibles)?	mg/mmol créatinine mg/g créatinine μg/min mg/24h μg/mL mg/L
41. Quelles sont vos valeurs de référence (cut-off) pour l’albumine urinaire?	
IDMS - isotope dilution mass spectrometry. MDRD - modification of diet in renal disease. CKD-EPI - chronic kidney disease epidemiology collaboration. CAPA - Caucasian, Asian, pediatric and adult.	

**Table 4:** A proposed IFCC C-KD universal questionaire – Italian translation

**Domande**	**Opzioni di risposta**
**Creatinina plasmatica/sierica**	
1. Il suo laboratorio effettua la determinazione della creatinina plasmatica/s.?	Si No
2. Quale metodo analitico è usato nel suo laboratorio (è possibile selezionare più di una opzione di risposta)	Jaffe non compensata Jaffe compensata, riferibile IDMS Enzimatica, riferibile IDMS Su sangue intero (POCT) Chimica secca Altro (specificare)
3. Indicare la ditta che commercializza il kit diagnostico	
4. Indicare il tipo di strumento analitico usato	
5. Quale unità di misura compare nel referto del suo laboratorio?	mg/dL μmol/L doppia unità (entrambe)
6. Indicare gli intervalli di riferimento riportati nel referto per l’adulto	
***e*GFR_creatinina_**	
7. Nel suo referto, è riportato in modo automatico il valore di stima del filtrato glomerulare mediante formula (eGFR_creatinina_)?	Si, in modo automatico per ogni risultato di creatinina plasmatica/sierica Si, solo se richiesto dal medico prescrittore No
8. Ritiene sia fondamentale riportare sempre nel referto il valore di eGFR?	Si No
9. Quale formula applica per calcolare il valore *e*GFR_creatinina_ riportato nel referto (è possibile selezionare più di una opzione di risposta)	MDRD-4 per metodi non riferibili MDRD-4 per metodi riferibili MDRD-6 CKD-EPI_creatinina_ Cockroft-Gault Altro (specificare) Non so
10. Quale cut-off ha scelto per refertare *e*GFR_creatinina_ maggiore di (>) (esempio: *e*GFR>90) oppure ha scelto di refertare il risultato reale ottenuto dal calcolo (esempio: *e*GFR = 72 mL/min/1.73m^2^)?	Refertiamo il risultato reale esatto *e*GFR_creatinina_ ottenuto dalla formula Refertiamo *e*GFR > 60 mL/min/1.73m^2^ per qualsiasi risultato > 60 Refertiamo *e*GFR > 60 mL/min/1.73m^2^ per qualsiasi risultato > 90 Altro (specificare)
11. Come considera nel referto la variabile etnica (razza)?	Indicando solo 2 opzioni: caucasico o non caucasico La variabile etnica è inserita nel LIS al momento della richiesta Il paziente o il medico prescrittore compilano un modulo cartaceo La variabile etnica non è presa in considerazione Altro (specificare)
12. Nel referto sono inclusi commenti che descrivono possibili interferenze sui risultati di *e*GFR_creatinina_ in particolari condizioni fisiologiche? (è possibile selezionare più di una opzione di risposta)	Stati nutrizionali Gravidanza Ricovero ospedalero Nessun commento/osservazione Altro (specificare)
13. Condizioni particolari: paziente in ricovero ospedaliero	Refertiamo *e*GFR_creatinina_ nelle modalità sopra esposte Non refertiamo *e*GFR_creatinina_
14. Quale formula applica per calcolare *e*GFR in età pediatrica?	Formula di Schwartz originale Formula di Schwartz modificata Altro (specificare) Non calcoliamo e non refertiamo *e*GFR_creatinina_ in età pediatrica
**Cistatina C (siero/plasma)**	
15. Nel suo laboratorio è eseguita la determinazione della cistatina C?	Si No
16. Quale metodo analitico è usato nel suo laboratorio (è possibile selezionare più di una opzione di risposta)	Immunoturbidimetrico Immunonefelometrico Altro (specificare)
17. Specificare la riferibiltà (traceability) del/i metodo/i usati nel suo laboratorio	Riferibile allo standard calibratore primario Non riferibile
18. Indicare la ditta che commercializza il kit diagnostico	
19. Indicare il tipo di strumento analitico usato	
20. Quale unità di misura compare nel referto del suo laboratorio?	mg/L Altro (specificare)
21. Indicare l’intervallo di riferimento usato per gli adulti nel suo laboratorio	Uomini: Donne: Intervallo unico:
***e*GFR_cistatina C_**	
22. Nel suo referto, insieme al risultato della cistatina C è riportato in modo automatico il valore di stima del filtrato glomerulare mediante formula (eGFR_cistatina C_) ?	Si, in modo automatico per ogni risultato di cistatina C plasmatica/sierica Si, solo se richiesto dal medico prescrittore No
23. Pensa sia importante introdurre nel referto il valore di eGFR_cistatina C_?	Si No Nel nostro laboratorio lo abbiamo già introdotto
24. Quale formula applica per calcolare il valore *e*GFR_cistatina C_ riportato nel referto (è possibile selezionare più di una opzione di risposta)	CAPA CKD-EPI_cistatina C_ Altro (specificare)
25. Quale cut-off ha scelto per refertare *e*GFR_cistatina C_ maggiore di (>) (esempio: *e*GFR > 90) oppure ha scelto di refertare il risultato reale ottenuto dal calcolo (esempio: *e*GFR = 72 mL/min/1.73 m^2^)?	Refertiamo il risultato reale esatto *e*GFR_cistatina C_ ottenuto dalla formula Refertiamo *e*GFR > 60 mL/min/1.73m^2^ per qualsiasi risultato > 60 Refertiamo *e*GFR > 60 mL/min/1.73m^2^ per qualsiasi risultato > 90 Altro (specificare)
26. Quale formula applica per calcolare *e*GFR_cistatina C_ in età pediatrica?	CAPA CKD-EPI_cistatina C_ Altro (specificare) Non calcoliamo e non refertiamo *e*GFR_cistatina C_ in età pediatrica
**Proteinuria e albuminuria**	
27. Nel suo laboratorio quali degli esami indicati a destra si eseguono?	Proteinuria Albuminuria Entrambi Nessuno dei 2
28. Nel suo laboratorio, qual è il tipo di campione raccomandato per eseguire la determinazione della proteinuria?	Raccolta delle 24 ore Campione estemporaneo Prima minzione del mattino Non specificato (nessuna raccomandazione) Altro (specificare)
29. Quale metodo analitico è usato nel suo laboratorio per la determinazione della proteinuria?	Nefelometrico Turbidimetrico Colorimetrico Chimica secca Altro (specificare)
30. Specificare la riferibiltà (traceability) del/i metodo/i usati nel suo laboratorio	Riferibile allo standard calibratore primario Non riferibile
31. Indicare la ditta che commercializza il kit diagnostico	
32. Indicare il tipo di strumento analitico usato	
33. Quale unità di misura compare nel referto del suo laboratorio per la proteinuria (è possibile selezionare più di una opzione di risposta)?	mg/mmol di creatinina mg/g di creatinina g/24h g/L
34. Indicare gli intervalli di riferimento riportati nel referto per la proteinuria	
35. Nel suo laboratorio, qual è il tipo di campione raccomandato per eseguire la determinazione dell’albuminuria?	Raccolta delle 24 ore Campione estemporaneo Prima minzione del mattino Non specificato (nessuna raccomandazione) Altro (specificare)
36. Quale metodo analitico è usato nel suo laboratorio per la determinazione dell’albuminuria?	Nefelometrico Turbidimetrico Colorimetrico Chimica secca Altro (specificare)
37. Specificare la riferibiltà (traceability) del/i metodo/i usati nel suo laboratorio	Riferibile allo standard calibratore primario Non riferibile
38. Indicare la ditta che commercializza il kit diagnostico	
39. Indicare il tipo di strumento analitico usato	
40. Quale unità di misura compare nel referto del suo laboratorio per la proteinuria (è possibile selezionare più di una opzione di risposta)?	mg/mmol di creatinina mg/g di creatinina μg/min mg/24h μg/mL mg/L
41. Indicare gli intervalli di riferimento riportati nel referto per l’albuminuria	
IDMS - isotope dilution mass spectrometry. MDRD - modification of diet in renal disease. CKD-EPI - chronic kidney disease epidemiology collaboration. CAPA - Caucasian, Asian, pediatric and adult.	

**Table 5:** A proposed IFCC C-KD universal questionaire – Spanish translation

**Preguntas**	**Respuestas**
Creatinina en suero	
1. Su laboratorio mide creatinina en suero?	si no
2. Por favor, especifique su método de medida de creatinina en suero(puede mencionar más de uno)	Jaffe no compensado Jaffe compensado, calibración IDMS método enzimático referidos al método de IDMS creatinina en sangre total. química seca otros (por favor, especifique)
3. Especifique su fabricante(s) de reactivos	
4. Especifique su tipo de instrumento(s)	
5. En qué unidades reporta los resultados de creatinina en suero?	mg/dL µmol/L ambos
6. Especifique el intervalo de referencia de creatinina en suero para adultos usado en su laboratorio	
eGFR_creatinine_	
7. Usted reporta automaticamente los resultados del índice estimado de filtración glomerular de creatinina (eGFR_creatinine_) junto con la concentración de la creatinina sérica?	si, automáticamente con cada resultado de creatinina sérica sí, siempre que sea ordenado por el médico no
8. Ud. considera informar el eGFR_creatinine_?	si no
9. Qué ecuación usa para estimar el indice de filtración glomerular para un informe de rutina? (puede seleccionar más de uno)	MDRD-4 ecuación para creatinina no estandarizada MDRD-4 ecuación para creatinina estandarizada MDRD-6 CKD-EPI ecuación Cockroft-Gault Otros (por favor, especifique)
10. A partir de qué valor Ud. informa eGFR_creatinine_ mayor que (>)?	Se informan todos los valores de eGFR_creatinine_ como: el número exacto. Se informan valores por encima de 60 mL/min/1.73m^2^ como: > 60 mL/min/1.73m^2^ Se reportan valores por encima de 90 mL/min/1.73m^2^ como: > 90 mL/min/1.73m^2^ Otros (por favor, especifique)
11. Cómo informa los resultados del parámetro “raza“?	dos resultados, “Negro“ y “Blanco“ durante la consulta determina la raza se pregunta al paciente para informar su raza el parámetro “Raza“no está incluído otros (por favor, especifique)
12. En el resultado de eGFR_creatinine_ están incluídas observaciones y posibles variaciones de este valor con:	estado nutricional embarazo pacientes ingresados no se incluyen observaciones acerca de posibles variaciones de eGFR otros (por favor, especifique)
13. Situaciones especiales: pacientes ingresados	Se informa eGFR_creatinine_ para pacientes ingresados No se informan eGFR_creatinine_ para pacientes ingresados
14. Qué ecuaciones /fórmulas usa Ud. para estimar la GFR en niños?	Ecuación de Schwartz (original) Ecuación de Schwartz (modificada) otros(por favor, especifique) No se calcula la eGFR_creatinine_ para niños
**Cistatina C en suero (plasma)**	
15. Su laboratorio mide cistatina C en suero?	si no
16. Por favor, identifique su método para medir cistatina C en suero (puede seleccionar más de uno)	inmunoturbidimetría innmunonefelometría otros (por favour, especificar)
17. Especificar la trazabilidad de su método(s)	
18. Especificar el fabricante de su reactivo(s)	
19. Especificar su tipo de instrumento (s)	
20. En qué unidades son informados sus resultados de cistatina C en suero?	mg/L otros (por favor, especifique)
21.Especificar el intervalo de referencia para adultos de cistatina C en su laboratorio	
**eGFR_cystatin_**_C_	
22. Reporta Ud. automaticamente los resultados del indice de Filtración Glomerular de cistatina C (eGFR_cystatin C_) junto con la concentración de cistatina C en suero?	si, automaticamente con cada resultado de cistatina C en suero. Si, siempre que lo ordene el médico. no
23. Considera reportar el eGFR_cystatin C_?	si no
24. Cual ecuación para adultos de eGFR_cystatin C_ usa Ud. para el informe de rutina? (puede elegir más de una)	CAPA CKD-EPI_cystatin C_ Otros (por favor, especifique)
25. ¿A partir de qué valor Ud. informa una eGFR_cystatin C_ mayor que(>)?	se informan todos los valores para eGFR_cystatin C_ como un número exacto. Se informan valores por encima de 60 mL/min/1.73m^2^ como > 60 mL/min/1.73m^2^ Se informan valores por encima de 90 mL/min/1.73m^2^ como > 90 mL/min/1.73m^2^ otros (por favor, especifique)
26. Qué ecuación de eGFR_cystatin C_-usa para estimar la GFR en niños?	CAPA CKD-EPI_cystatin C_ Otros (por favor, especifique) no se estima GFR para niños
**Proteina en orina y albumina**	
27. Ud. hace los siguientes tests?	proteina en orina albúmina en orina ninguna de estas
28. Qué muestra es recomendada para medir proteína en orina?	muestra de orina de 24 horas. muestra de orina al azar. primera orina de la mañana no especificada otras (por favor, especifique)
29. Por favor, especificar su método para medir proteína urinaria.	nefelometría turbidimetría colorimetría química seca otros (por favor, especifique)
30. Especificar la trazabilidad de su(s) método(s)	
31. Especificar la compañía fabricante de su reactivo(s)	
32. Especificar el tipo de su instrumento(s)	
33. Como informa los resultados de proteína en orina? (puede seleccionar más de una)	mg/mmol creatinina mg/g creatinina g/24h g/L
34. Por favor, especificar su intervalo de referencia (valor cut-off) para proteínas en orina.	
35. Cuál de las muestras es recomendada para medir albúmina en orina?	muestra de orina de 24 hs muestra de orina al azar muestra de primera orina de la mañana no especificada otros (por favor, especifique)
36. Por favor, especificar su método para medir albúmina en orina.	nefelometría turbidimetría colorimetría química seca otros (por favor, especifique)
37. Especificar la trazabilidad de su método(s)	
38. Especificar el fabricante de su reactivo(s)	
39. Especificar su tipo de instrumento(s)	
40. Como informar los resultados de albúmina en orina? (puede elegirse más de uno)	mg/mmol creatinina mg/g creatinina μg/min mg/24h μg/mL mg/L
41.Por favor, especificar su intervalo de referencia (valor cut-off) para la albumina en orina	
IDMS - isotope dilution mass spectrometry. MDRD - modification of diet in renal disease. CKD-EPI - chronic kidney disease epidemiology collaboration. CAPA - Caucasian, Asian, pediatric and adult.	

**Table 6:** A proposed IFCC C-KD universal questionaire – Portuguese translation

**Perguntas**	**Respostas**
**Creatinina Sérica**	
1. O seu Laboratório mede a creatinine sérica?	Sim Não
2. Por favor especifique o método que utiliza para medir a creatinine sérica? (mais do que pode ser seleccionado)	Jaffe Não Compensado Jaffe Compensado, IDMS Calibrado Método Enzimático IDMS ratreável Creatinina em sangue total Química Seca Outro (Por favor, especifique)
3. Especifique o seu fabricante de Reagente(s)	
4. Especifique o seu Autoanalizador	
5. Em que unidades a creatinine sérica é reportada?	mg/dL µmol/L Ambas
6. Especifique o intervalo de referencia para adultos utilizado para a creatinine sérica no seu Laboratório	
**TFGE (Taxa de Filtração Glomerular estimada)**	
7. Reporta automaticamente os resultados de TFGe cada vez que reporta a creatinina sérica?	Sim, automaticaemnte com cada resultado de creatinine sérica Sim, quando solicitado pelo Clínico Não
8. Considera reporter a TFGe?	Sim Não
9. Qual a equação que utiliza na rotina para estimar a TFG (mais do que uma po der seleccionada)	Equação MDRD-4 para creatinine não Equação MDRD-4 para creatinine padronizada Equação MDRD-6 Equação CKD-EPI Equação Cockroft-Gault Outra (Por favor, especifique)
10. Para que valor coloca TFGe superior a (>)?	Reportamos sempre um número exacto Reportamos valores superiores a 60 mL/min/1.73m^2^ como > 60 mL/min/1.73m^2^ Reportamos valores superiores a 90 mL/min/1.73m^2^ como > 90 mL/min/1.73m^2^ Outro (Por favor, especifique)
11. Como reporta os resultados de acordo com o parametro “raça”?	Dois resultados, “Negra” e “Branca” Raça avaliada durante a consulta O doente é questionado para reporter seua raça Parametro “Raça” não é incluído Outro (Por favor, especifique)
12. No resultado de TFGe são incluídas observações e variações possíveis do valor com:	Estado Nutricional Gravidez Doentes Internados Não incluímos observações sobre possíveis variações da TFGe Outro(s) (Por favor, especifique)
13. Situações Especiais: Doentes Internados	Reportamos TFGe para doentes internados Não Reportamos TFGe para doentes internados
14. Que Equações/Fórmulas utiliza para estimar TFG em crianças?	Equação de Schwartz (original) Equação de Schwartz (modificada) Outra (Por favor, especifique) Não calculamos TFGe para crianças
**Cistatina C sérica (plasma)**	
15. O seu Laboratório mede a Cistatina C?	Sim Não
16. Por favor, especifique o seu método para medição de Cistatina C sérica (mais do que pode ser seleccionado)	Imunoturbidimetria imunonefelometria Outro (Por favor, especifique)
17. Especifique o método rastreável	
18. Especifique o fabricante de reagent(s)	
19. Especifique o seu autoanalizador(s)	
20. Em que unidades os valores de Cistatina C são reportados?	mg/L Outro (Por favor, especifique)
21. Especifique intervalo de referência para adultos utilizado para a Cistatina C sérica no seu laboratório	
**Taxa de Filtração Glomerular estimada por Cistatina C (TFGe Cys)**	
22. Reporta automaticamente os resultados de TFGe Cys cada vez que é reportado o valor da Cistatina C sérica?	Sim, automaticamente com cada resultado de Cistatina C Sérica Sim, quando solicitado pelo Clínico Não
23. Considera reporter a TFGe Cys?	Sim Não
24. Que Equação TFGe Cys para adultos utiliza na rotina (mais do que uma pode ser seleccionada)	CAPA CKD-EPI_cystatin C_ Outra (Por favor, especifique)
25. Em que valor coloca TFGe Cys superior a (>)?	Reportamos todos os valores de TFGe Cys como número exacto Reportamos valores superiors a 60 mL/min/1.73m^2^ como > 60 mL/min/1.73m^2^ Reportamos valores superiors a 90 mL/min/1.73m^2^ como > 90 mL/min/1.73m^2^ Outra (Por favor, especifique)
26. Que Equação para TFGe Cys utiliza para estimar TFG em crianças?	CAPA CKD-EPI_cystatin C_ Outra (Por favor, especifique) Não estimamos TFGe Cys para crianças
**Proteinúria e Albuminúria**	
27. Efectua os seguintes Testes?	Proteinúria Albuminúria Nenhum deles
28. Qual é amostra recomendada para medir proteinúria?	Amostra de urina de 24 horas Amostra de Urina Aleatória Amostra da 1ª Urina da manhã Não especificado Outra (Por favor, especifique)
29. Por favor especifique o método que utiliza para medir a proteinúria?	Nefelometria Turbidimetria Colorimetria Química Seca Outro (Por favor, especifique)
30. Especifique o método rastreável	
31. Especifique o fabricante de reagent(s)	
32. Especifique o seu autoanalizador(s)	
33. Como reporta os resultados de proteinuria (mais do que um pode ser seleccionado)?	mg/mmol creatinina mg/g creatinina g/24h g/L
34. Por favor especifique interevalo de referência (valor de Cut-Off) para a Proteinúria	
35. Qual é a amostra recomendada para medir Albuminúria ?	Amostra de urina de 24 horas Amostra de Urina Aleatória Amostra da 1ª Urina da manhã Não especificado Outra (Por favor, especifique)
36. Por favor especifique o método que utiliza para medir a Albuminúria?	Nefelometria Turbidimetria Colorimetria Química Seca Outro (Por favor, especifique)
37. Especifique o método rastreável	
38. Especifique o fabricante de reagent(s)	
39. Especifique o seu autoanalizador(s)	
40. Como reporta resultados de Albuminúria (mais do que um pode ser seleccionado)?	mg/mmol creatinina mg/g creatinina μg/min mg/24h μg/mL mg/L
41. Por favor especifique interevalo de referência (valor de Cut-Off) para a Albuminúria	
IDMS - isotope dilution mass spectrometry. MDRD - modification of diet in renal disease. CKD-EPI - chronic kidney disease epidemiology collaboration. CAPA - Caucasian, Asian, pediatric and adult.	

**Table 7:** A proposed IFCC C-KD universal questionaire – Turkish translation

**Sorular**	**Cevaplar**
**Serum kreatinini**	
1. Laboratuvarınızda serum kreatinini ölçülüyor mu?	evet hayır
2. Lütfen serum kreatinini için ölçüm yönteminizi belirtiniz (birden fazla seçenek işaretlenebilir)	kompanse olmayan Jaffe kompanse Jaffe, IDMS’e göre kalibre IDMS yöntemiyle uyumlu enzimatik yöntem tam kanda kreatinin kuru kimya diğer (lütfen belirtiniz)
3. Kullandığınız ayıracın üretici(ler)ini belirtiniz	
4. Kullandığınız cihaz(lar)ı belirtiniz	
5. Serum kreatinini hangi birimle rapor ediyorsunuz?	mg/dL µmol/L her ikisi de
6. Laboratuvarınızda erişkinlerde serum kreatinini için kullanılan referans aralığını belirtiniz	
eGFR_kreatinin_	
7. Serum kreatinini ile birlikte tahmini Glomerüler Filtrasyon Hızını (eGFR_kreatinin_) otomatik olarak rapor ediyor musunuz?	evet, serum kreatinini ile birlikte otomatik olarak her zaman evet, klinisyen tarafından istendiğinde hayır
8. eGFR_kreatinin_’in rapor edilmesini önemli görüyor musunuz?	evet hayır
9. Rutin çalışmalarda GFR hesabı için hangi formülü kullanıyorsunuz? (birden fazla seçenek işaretlenebilir)	standardize olmayan kreatinin için MDRD-4 formülü standardize kreatinin için MDRD-4 formülü MDRD-6 CKD-EPI formülü Cockroft-Gault diğer (lütfen beirtiniz) bilmiyorum
10. eGFR_kreatinin_ sonucunu hangi değere kadar rapor ediyorsunuz?	tüm eGFR_kreatinin_ sonuıçlarını rakam olarak rapor ediyoruz 60 mL/min/1.73m^2^ den büyük sonuçları > 60 mL/min/1.73m^2^ olarak rapor ediyoruz 90 mL/min/1.73m^2^ den büyük sonuçları > 90 mL/min/1.73m^2^ olarak rapor ediyoruz diğer (lütfen belirtiniz)
11. „Irk“ parametresi ile ilgili sonuçları nasıl rapor ediyorsunuz?	„Siyah“ ve „Beyaz“ olarak ırk randevu sırasında değerlendiriliyor hastaya ırkını belirtmesi rica ediliyor „Irk“ parametresi yer almıyor diğer (lütfen belirtiniz)
12. eGFR_kreatinine_ sonucunda, bu değer ile birlikte hangi gözlemler veya olası değişkenler yer alıyor?	beslenme durumu gebelik kabul edilen hastalar gözlemler ve olası eGFR değişkenleri yer almıyor diğer (lütfen belirtiniz)
13. Özel durumlar: yatan hastalar	eGFR_kreatinin_’i yatan hastalarda rapor ediyoruz eGFR_kreatinin_’i yatan hastalarda rapor etmiyoruz
14. Çocuklarda GFR için hangi denklemi/formülü kullanıyorsunuz?	Schwartz formülü (orijinal) Schwartz formülü (değişik) diğer (lütfen belirtiniz) Çocuklarda eGFR_kreatinin_’i hesaplamıyoruz
**Serum (plazma) cystatin C**	
15. Laboratuvarınızda serum cystatin C ölçülüyor mu?	evet hayır
16. Lütfen serum cystatin C ölçüm yönteminizi belirtiniz (birden fazla seçenek işaretlenebilir)	immünotürbidimetri immünonefelometri diğer (lütfen belirtiniz)
17. Kullandığınız yöntemin izlenebilirliğini belirtiniz	
18. Kullandığınız ayıracın üretici(ler)ini belirtiniz	
19. Kullandığınız cihaz(lar)ı belirtiniz	
20. Serumda cystatin C’yi hangi birimle rapor ediyorsunuz?	mg/L diğer (lütfen belirtiniz)
21. Laboratuvarıızda kullanılan erişkin cystatin referans aralığını belirtiniz	
**eGFR_cystatin_**_C_	
22. Serum cystatin C konsantrasyonu ile birlikte tahmini Glomerüler Filtrasyon Hızını (eGFR_cystatin C_) otomatik olarak rapor ediyor musunuz?	evet, serum cystatin C ile birlikte otomatik olarak her zaman evet, klinisyen tarafından istendiğinde hayır
23. eGFR_cystatin C_’nin rapor edilmesini önemli görüyor musunuz?	evet hayır her zaman rapor ediyoruz
24. Rutin çalışmalarda erişkinler için hangi eGFR_cystatin C_ denklemini kullanıyorsunuz? (birden fazla seçenek işaretlenebilir)	CAPA CKD-EPI_cystatin C_ diğer (lütfen belirtiniz)
25. eGFR_cystatin C_ sonucunu hangi değere kadar rapor ediyorsunuz?	tüm eGFR_cystatin C_ sonuıçlarını rakam olarak rapor ediyoruz 60 mL/min/1.73m^2^ den büyük sonuçları > 60 mL/min/1.73m^2^ olarak rapor ediyoruz 90 mL/min/1.73 m^2^ den büyük sonuçları > 90 mL/min/1.73m^2^ olarak rapor ediyoruz diğer (lütfen belirtiniz)
26. Çocuklarda hangi eGFR_cystatin C_-denklemini kullanıyorsunuz?	CAPA CKD-EPI_cystatin C_ diğer (lütfen belirtiniz) Çocuklarda GFR sonucu vermiyoruz
**İdrar protein and albümini**	
27. Aşağıdaki testleri yapıyor musunuz?	idrar proteini idrar albümini her ikisi de hiçbiri
28. İdrar proteini ölçümünde hangi numune önerilir?	24 saatlik idrar Rastgele (random) idrar Sabah ilk idrar belli değil diğer (lütfen belirtiniz)
29. Lütfen idrar proteini ölçüm yönteminizi belirtiniz	nefelometri türbidimetri kolorimetri kuru kimya diğer (lütfen belirtiniz)
30. Kullandığınız yöntemin izlenebilirliğini belirtiniz	
31. Kullandığınız ayıracın üreticisini belirtiniz	
32. Kullandığınız cihaz(lar)ı belirtiniz	
33. İdrar proteinini nasıl rapor ediyorsunuz? (birden fazla seçenek işaretlenebilir)	mg/mmol kreatinin mg/g kreatinin g/24 saat g/L
34. Lütfen idrar protein için kullandığınız referans aralığını (kesim değerini) belirtiniz	
35. İdrarda albümin ölçümü için hangi numune önerilir?	24 saatlik idrar rastgele (random) idrar sabah ilk idrar belli değil diğer (lütfen belirtiniz)
36. Lütfen idrar albümini için kullandığınız ölçüm yöntemini belirtiniz	nefelometri türbidimetri kolorimetri kuru kimya diğer (lütfen belirtiniz)
37. Kullandığınız yöntemin izlenebilirliğini belirtiniz	
38. Kullandığınız ayıracın üreticisini belirtiniz	
39. Kullandığınız cihaz(lar)ı belirtiniz	
40. İdrarda albümin sonuçlarını nasıl rapor ediyorsunuz? (birden fazla seçenek işaretlenebilir)	mg/mmol kreatinin mg/g kreatinin μg/dakika mg/24 saat μg/mL mg/L
41. Lütfen idrar albümini için kullandığınız referans aralığını (kesim değerini) belirtiniz	
IDMS - isotope dilution mass spectrometry. MDRD - modification of diet in renal disease. CKD-EPI - chronic kidney disease epidemiology collaboration. CAPA - Caucasian, Asian, pediatric and adult.	

**Table 8:** A proposed IFCC C-KD universal questionaire – Chinese translation

问题	答案
血清肌酐	
1. 你的实验室测量血清肌酐吗?	是 否
2. 请说明你的实验室测量血清肌酐的方法 (可多选)	全血肌酐 干化学法 其他(请说明)
3. 你的试剂制造商	
4. 你的仪器型号	
5. 血清肌酐的报告单位	毫克/分升 微摩尔/升 两者都有
6. 请说明在你的实验室中,成人血清肌酐的参考范围	
估算的肾小球滤过率	
7. 在报告血清肌酐值时,你是否会自动报告估算的肾小球滤过率(eGFR)?	是,会自动同时报告 只当医生要求时 不会
8. 你是否会考虑报告估算的肾小球滤过率(eGFR)?	是 否
9. 你使用哪条公式来估算肾小球滤过率(可多选)	非标准化肌酐值用MDRD-4公式 标准化肌酐值用MDRD-4公式 MDRD-6 CKD-EPI 公式 Cockroft-Gault 其他(请说明)
10. 当eGFR高于何值时,会报告(>)?	我们报告实际的eGFR值 当eGFR高于60 mL/min/1.73 m^2^时,我们报告 > 60 mL/min/1.73 m^2^ 当eGFR高于90 mL/min/1.73 m^2^时,我们报告 > 90 mL/min/1.73 m^2^ 其他(请说明)
11. 如何报告与「种族」相关的参数?	两个结果,「黑人」和「白人」 在预约时评估其种族 问病人的种族 没有包括不同种族的结果 其他(请说明)
12. 在报告eGFR时,是否包含一些会影响eGFR值的情况	营养状态 妊娠 住院病人 报告没有包括一些会影响eGFR值的情况 其他(请说明)
13. 特殊情况:住院病人	我们会报告住院病人的eGFR值 我们不会报告住院病人的eGFR值
14. 你使用哪条公式来估算儿童肾小球滤过率?	Schwartz公式 (原始) Schwartz公式 (修改) 其他(请说明) 我们不会估算儿童肾小球滤过率
血清半胱氨酸蛋白酶抑制剂C	
15. 你的实验室测量血清半胱氨酸蛋白酶抑制剂C吗?	是 否
16. 请说明你的实验室测量血清半胱氨酸蛋白酶抑制剂C的方法 (可多选)	免疫透射比浊法 免疫散射比浊法 其他(请说明)
17. 说明你的方法溯源性	
18. 说明你的试剂制造商	
19. 说明你的仪器型号	
20. 血清半胱氨酸蛋白酶抑制剂C的报告单位	毫克/升 其他(请说明)
21. 请说明在你的实验室中,成人血清半胱氨酸蛋白酶抑制剂C的参考范围	
估算的肾小球滤过率_半胱氨酸蛋白酶抑制剂C_	
22. 在报告血清半胱氨酸蛋白酶抑制剂C的浓度时,你是否会自动报告估算肾小球滤过率_半胱氨酸蛋白酶抑制剂C_ (eGFR_cystatin C_)?	是,会自动同时报告 只当医生要求时 不会
23. 你是否会考虑报告估算肾小球滤过率_半胱氨酸蛋白酶抑制剂C_ (eGFR_cystatin C_)?	是 否
24. 你常规使用哪条公式来计算成人的估算肾小球滤过率_半胱氨酸蛋白酶抑制剂C_ (eGFR_cystatin C_) (可多选)	CAPA CKD-EPI_cystatin C_ 其他(请说明)
25. 当估算肾小球滤过率_半胱氨酸蛋白酶抑制剂C_ (eGFR_cystatin C_)高于何值时,会报告(>)?	我们报告实际的估算肾小球滤过率_半胱氨酸蛋白酶抑制剂C_ (eGFR_cystatin C_)的值 当eGFR_cystatin C_高于60 mL/min/1.73 m^2^时,我们报告 > 60 mL/min/1.73 m^2^ 当eGFR_cystatin C_高于90 mL/min/1.73 m^2^时,我们报告 > 90 mL/min/1.73 m^2^ 其他(请说明)
26. 你使用哪条公式来计算儿童的估算肾小球滤过率_半胱氨酸蛋白酶抑制剂C_ ?	CAPA CKD-EPI_cystatin C_ 其他(请说明) 在儿童中,我们不估算肾小球滤过率
尿蛋白和尿白蛋白	
27. 你们有没有做以下的检验项目?	尿蛋白 尿白蛋白 以上两者都没有
28. 你们推荐用哪种样本来测量尿蛋白?	24小时尿 随机尿 晨起第一次尿 没有特定 其他(请说明)
29. 请说明检测尿蛋白的方法	散射比浊法 透射比浊法 比色法 干化学法 其他(请说明)
30. 说明你的方法溯源性	
31. 说明你的试剂制造商	
32. 说明你的仪器型号	
33. 你如何报告尿蛋白的结果(可多选)?	毫克/毫摩尔肌酐 毫克/克肌酐 克/24小时 克/升
34. 请说明你的尿蛋白参考范围	
35. 你们推荐用哪种样本来测量尿白蛋白?	24小时尿 随机尿 晨起第一次尿 没有特定 其他(请说明)
36. 你们用哪种方法来测量尿白蛋白?	散射比浊法 透射比浊法 比色法 干化学法 其他(请说明)
37. 说明你的方法溯源性	
38. 说明你的试剂制造商	
39. 说明你的仪器型号	
40. 你如何报告尿白蛋白的结果(可多选)?	毫克/毫摩尔肌酐 毫克/克肌酐 微克/分钟 毫克/24小时 微克/毫升 毫克/升
41. 请说明你的尿白蛋白参考范围	
MDRD - modification of diet in renal disease. CKD-EPI - chronic kidney disease epidemiology collaboration. CAPA - Caucasian, Asian, pediatric and adult.	

**Table 9:** A proposed IFCC C-KD universal questionaire – Russian translation

**Вопрос**	**ответ**
**Креатинин сыворотки**	
1. Ваша лаборатория измеряет креатинин сыворотки?	да нет
2. Пожалуйста, определите свой метод для измерения креатинина сыворотки (можете выбрат больше чем один ответ)	неданный компенсацию Jaffe данный компенсацию Jaffe, IDMS калибрована ферментативный метод выровнен к методу IDMS креатинин цельной крови сухая химия другой (пожалуйста, укажите)
3. Укажите своего производителя (производителей) реактива	
4. Укажите свой тип инструмента (инструментов)	
5. В каких единицах сообщают о результатах креатинина сыворотки?	mg/dL µmol/L обе
6. Укажите справочный интервал для взрослых, используемый для креатинина сыворотки в Вашей лаборатории	
**eGFR_creatinine_**	
7. Вы автоматически сообщаете о результатах предполагаемой Скорости клубочковой фильтрации (eGFRcreatinine) наряду с концентрацией креатинина сыворотки?	да, автоматически с каждым результатом креатинина сыворотки да, каждый раз, когда заказал врач нет
8. Вы рассматриваете создание отчетов о eGFRcreatinine?	да нет
9. Какое уравнение используете Вы для оценки GFR для создания отчетов режима (можно выбрать больше чем один ответ)	Уравнение MDRD-4 для нестандартизированного креатинина Уравнение MDRD-4 для стандартизированного креатинина MDRD-6 Уравнение CKD-ЭПИТАКСИАЛЬНОГО-СЛОЯ Кокрофт-Голт другой (пожалуйста, укажите), Я не знаю
10. На какой отметке Вы указываете повишение eGFRкреатинин (>)?	указываем все отметки eGFR_creatinine_ в точных цифрах указываем отметки выше 60 mL/min/1.73m^2^ as >60 mL/min/1.73m^2^ указываем отметки выше 90 mL/min/1.73m^2^ as >90 mL/min/1.73m^2^ другие (укажите)
11. Как Вы сообщаете о результатах относительно параметра „раса“?	два результата, „Темнокожий “и „Белый “ раса оценена во время назначения пациента просят сообщить о его расе параметр „Раса“ не включен другой (укажите)
12. В результате eGFRcreatinine включены наблюдения и возможные изменения этого значения с (несколько возможных ответов):	состояние питания беременность информации полученые от пациента мы не включаем наблюдения о возможных изменениях eGFR другой (пожалуйста, укажите)
13. Специальные ситуации: стационарные больные	даем eGFR_creatinine_ для стационарных болбных не даем eGFR_creatinine_ для стационарных болбных
14. Какие уравнения/формулы Вы используете, чтобы оценить GFR у детей?	Уравнение Шварца (оригинальное) Уравнение Шварца (изменено) другой (пожалуйста, укажите), мы не измеряем eGFRcreatinine для детей
**Сыворотка (плазма) cystatin C**	
15. Ваша лаборатория измеряет сыворотку cystatin C?	да нет
16. Пожалуйста, укажите свой метод для измерения сыворотки cystatin C (можно больше чем один)	иммунонефелометрия иммунонефелометрия другой (пожалуйста, укажите)
17. Укажите метод (методы) отслеживаемости	
18. Укажите производителя реактива	
19. Какой инструмент Вы используете	
20. В каких единицах сыворотка измеряется cystatin C?	mg/L другие (укажите)
21. Определите справочный интервал для взрослых, используемых для сыворотки cystatin C в Вашей лаборатории	
**eGFR_cystatin_**_C_	
22. Вы автоматически сообщаете о результатах предполагаемой Скорости клубочковой фильтрации, основанной на cystatin C (eGFRcystatin C) наряду с сывороткой cystatin C концентрации?	да, автоматически с каждым результатом сыворотки cystatin C да, каждый раз, когда заказал врач нет
23. Вы рассматриваете создание отчетов о eGFRcystatin C?	да нет мы уже сообщаем о нем
24. Какое eGFRcystatin C-уравнение для взрослых используете Вы для создания рутинных отчетов (vozmo\en больше чем один ответ)	CAPA CKD-EPI_cystatin C_ какое (укажите)
25. На какой отметке Вы указываете повишение eGFRкреатинин (>)?	указываем все отметки eGFR_creatinine_ в точных цифрах указываем отметки выше 60 mL/min/1.73m^2^ as >60 mL/min/1.73m^2^ указываем отметки выше 90 mL/min/1.73m^2^ as >90 mL/min/1.73m^2^ другие (укажите)
26. Какие уравнения eGFR_cystatin C_- Вы используете, чтобы оценить GFR у детей?	CAPA CKD-EPI_cystatin C_ другие (укажите) не мерим GFR для детей
**Белок мочи и альбумин**	
27. Вы выполняете следующие анализы?	белок мочи альбумин мочи оба ни один из них
28. Какой образец рекомендуете для измерения белка мочи?	24-часовой образец мочи случайный образец мочи первый утренний образец мочи не определенный другой (пожалуйста, укажите)
29. Пожалуйста, укажите свой метод для измерения белка мочи	нефелометрия турбидиметрия колориметрия сухая химия другой (пожалуйста, укажите)
30. Укажите свой метод (методы) отслеживания	
31. Укажите производителя (производителей) реактива	
32. Укажите тип инструмента (инструментов)	
33. Как Вы сообщаете о результатах белка мочи (можете выбрать больше чем один ответ)?	mg/mmol креатинина mg/g креатинина g/24h g/L
34. Пожалуйста, укажите справочный интервал для белка мочи	
35. Какой образец рекомендуете для измерения альбумина мочи?	24-часовой образец мочи случайный образец мочи первый утренний образец мочи не определенный другой (пожалуйста, укажите)
36. Пожалуйста, укажите свой метод для измерения альбумина мочи	нефелометрия турбидиметрия колориметрия сухая химия другой (пожалуйста, укажите)
37. Укажите свой метод (методы) отслеживания	
38. Укажите производителя (производителей) реактива	
39. Укажите тип инструмента (инструментов)	
40. Как Вы сообщаете результатах альбумина мочи (можете выбрать больше чем один ответ)?	mg/mmol креатинина mg/g креатинина μg/min mg/24h μg/mL mg/L
41. Пожалуйста, укажите справочный интервал для альбумина мочи	
IDMS - isotope dilution mass spectrometry. MDRD - modification of diet in renal disease. CKD-EPI - chronic kidney disease epidemiology collaboration. CAPA - Caucasian, Asian, pediatric and adult	

In conclusion, we as an IFCC C-KD hope that this proposed questionnaire will be considered useful in assessing the current CKD status in many various national environments across the Globe.
